# Recombinant porcine rotavirus VP4 and VP4-LTB expressed in Lactobacillus casei induced mucosal and systemic antibody responses in mice

**DOI:** 10.1186/1471-2180-9-249

**Published:** 2009-12-04

**Authors:** Xinyuan Qiao, Guiwei Li, Xiangqing Wang, Xiaojing Li, Min Liu, Yijing Li

**Affiliations:** 1 Department of Preventive Veterinary, College of Veterinary, Northeast Agricultural University, 59 Mucai Street, Harbin, PR China; 2 College of Animal Science and Technology, Northeast Agricultural University, 59 Mucai Street, Harbin, PR China

## Abstract

**Background:**

Porcine rotavirus infection is a significant cause of morbidity and mortality in the swine industry necessitating the development of effective vaccines for the prevention of infection. Immune responses associated with protection are primarily mucosal in nature and induction of mucosal immunity is important for preventing porcine rotavirus infection.

**Results:**

*Lactobacillus casei *expressing the major protective antigen VP4 of porcine rotavirus (pPG612.1-VP4) or VP4-LTB (heat-labile toxin B subunit from *Echerichia coli*) (pPG612.1-VP4-LTB) fusion protein was used to immunize mice orally. The expression of recombinant pPG612.1-VP4 and pPG612.1-VP4-LTB was confirmed by SDS-PAGE and Western blot analysis and surface-displayed expression on *L. casei *was verified by immunofluorescence. Mice orally immunized with recombinant protein-expressing *L. casei *produced high levels of serum immunoglobulin G (IgG) and mucosal IgA. The IgA titters from mice immunized with pPG612.1-VP4-LTB were higher than titters from pPG612.1-VP4-immunized mice. The induced antibodies demonstrated neutralizing effects on RV infection.

**Conclusion:**

These results demonstrated that VP4 administered in the context of an *L. casei *expression system is an effective method for stimulating mucosal immunity and that LTB served to further stimulate mucosal immunity suggesting that this strategy can be adapted for use in pigs.

## Background

Rotaviruses are members of the family Reoviridae. Rotaviruses affecting pigs are classified as group A, B or C based on their respective inner capsid protein sequences[1]. The rotavirus double-stranded RNA genome is composed of 11 segments enclosed by a nonenveloped, triple-layered icosahedral capsid [2]. The outer capsid VP4 protein can induce neutralizing antibodies resulting in protecting herd from porcine rotavirus infection.

Porcine rotaviruses are the major cause of acute diarrhea in the piglets [3,4] and can cause mild-severe diarrhea associated with potentially high morbidity and mortality. Group A rotaviruses cause diarrhea in pigs both before and after weaning [5] and can account for 53 and 44% pre- and post-weaning rotavirus-associated diarrhea in swine, respectively [6]. A recent report attributed 89% of all rotavirus-associated diarrhea in commercial pig farms to group A rotavirus infections [7]. Since rotaviruses can survive in the environment for long period of time and are transmitted via the fecal-oral route outbreaks are difficult to control. Virion replication occurs at the tips of epithelial cell in intestinal villi and destroy enterocytes primarily in the jejunum and ileum resulting in villous atrophy [8,9]. Furthermore, nutrients cannot be digested or absorbed in the affected regions resulting in severe malabsorption [10]. A better understanding of rotavirus epidemiology will contribute to the optimization of current vaccines and prevention programs for the control of rotavirus infection. Currently available vaccines (mostly killed) can not offer efficient immunity. To stimulate efficient immunity, a large vaccine dose and repeated administration are usually required. This often results in undesirable clinical signs. To overcome these shortcomings, the potential development of lactic acid bacteria (LAB) to deliver heterologous antigen to the mucosal immune system has been proposed.

Since rotaviruses are enteric pathogens, mucosal immunity is likely to play an important role in protective immunity. Innate immune responses in gut provide the first line of defense against pathogenic microorganisms and also initiate acquired immune responses. Furthermore, immune responses resulting from oral immunization are the only suitable method of stimulating gut immunity [11] since this route facilitates stimulation of gut-associated lymphoid tissue (GALT) enhancing the production of anti-viral IgA [12].

Compared to recombinant antigens or heat-killed formulations, 'live' vaccines elicit the most effective protective responses since they stimulate both systemic and mucosal immunity [13-17]. However, oralvaccination presents a challenge since the gut milieu often denatures and/or inactivates potential vaccinogens therefore large vaccination doses and repeated vaccinations are required[18,19]. This often results in fecal shedding of the live vaccine in addition to causing fever and diarrhea [16,18,19]. These challenges can be overcome by using lactic acid bacteria (LAB) as antigen delivery system for the stimulation of mucosal immunity [20-25] owing to its safety. LAB are used in industrial food fermentation, preservation and have beneficial effects on the health of both humans and animals and 'generally regarded as safe, (GRAS'micro-organisms). In addition, many strains of LAB are able to survive and colonize the intestinal tract [26,27] inducing a non-specific immunoadjuvant effect [28] which prompted studies aimed at determining the oral vaccine potential of LAB-derived vaccines.

Since genetically engineered vaccines composed of a single recombinant antigen are poorly immunogenic, it is important to increase their immunogenicity by combining with appropriate adjuvants. The *E. coli *heat-labile toxin B subunit (LTB) has been shown to be a potent mucosal adjuvant [29-33] with low potential of eliciting allergic responses [34,35].

In this study, we tested the efficacy of the *L. casei *ATCC 393 expressing the heterologous VP4 porcine rotavirus protein and its ability acting as an antigen delivery system for oral vaccinations. We constructed recombinant strains expressing porcine rotavirus VP4 and VP4-LTB. The immunogenic potential of the two recombinant strains was analyzed after oral administration of live bacteria to mice. This is the first report describing the cloning and expression of porcine rotavirus genes in Lactobacillus. The data reported indicate that oral administration of two recombinant strains pPG612.1-VP4 or pPG612.1-VP4-LTB could induce specific anti-rotavirus mucosal and systemic immune responses. The potency of the immune responses measured was greater in animals immunized with *L. casei*-expressing the VP4-LTB fusion (compared to mice immunized with *L. casei *expressing VP4 only) demonstrating the efficacy of LTB as a mucosal adjuvant.

## Results

### Expression of VP4 and VP4-LTB in *L. casei*

The sequences of the respective *L. casei *393 transformants are confirmed by plasmid DNA sequencing and the result shows that there is no mutation in the transformants (data not shown).

**Figure 1:**
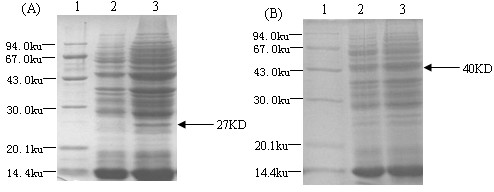
**Expression of VP4 and VP4-LTB in rLc393:pPG612.1-VP4 and pPG612.1-VP4-LTB**. Total cell lysates were analysed by SDS-PAGE. Coomassie blue gel staining shows the expression of a 27 KD and 40 KD fusion protein in lysates of rLc393 induced by xylose (Fig. 1A, lane 3 and Fig. 1B, lane 3), but not in basal MRS with glucose (Fig. 1A, lane 2 and Fig. 1B, lane 2).

**Figure 2:**
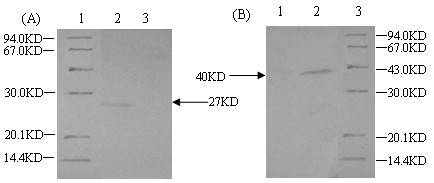
**Western-blotting analysis of VP4 and vp4-LTB expression in recombinant strain**. Immunoreactive bands were observed (Fig. 2A, lane 2 and Fig. 3B, lane 2) in the similar position as shown in the SDS-PAGE, however, there were no immunoblots in the same cell lysates induced by glucose (Fig. 2A, lane 3 and Fig. 3B, lane 1).

rLc393:pPG612.1-VP4 and pPG612.1-VP4-LTB were grown in basal MRS medium supplemented with either xylose or glucose. Cell lysates subjected to SDS-PAGE and showed the corresponding VP4 and VP4-LTB recombinant proteins at 27 and 40 kDa respectively after analyzing by Coomassie blue staining, following xylose induction (Figure 1A, lane 3 and Figure 1B, lane 3). Proteins were not expressed if cells were grown in basal MRS medium supplemented with glucose (Figure 1A, lane 2 and Figure 1B, lane 2). Gels run in parallel were transferred onto nitrocellulose membranes and examined by Western blot analysis using anti-VP4 antibodies. Immunoreactive bands corresponding to VP4 and VP4-LTB were observed at 27 and 40 kDa, respectively (Figure 2A, lane 2 and Figure 2B, lane 2). Reactive bands were not detected if the cells were instead grown in the presence of glucose (Figure 2A, lane 3 and Figure 2B, lane 1). These results demonstrated the efficiency and specificity of the *L. casei *xylose promoter.

### Immunofluorescence analysis

**Figure 3:**
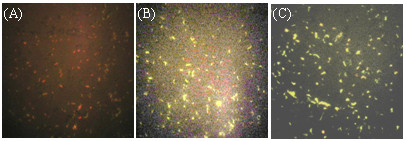
**Immunofluorescence analysis**. Wild-type L. casei 393 was induced by xylose, the result of immunofluorescence was negative, and the cells were dyed red by Evans blue (A). When pPG612.1-VP4 and pPG612.1-VP4-LTB were induced by xylose, there were green-yellow fluorescence reaction on the surface of the cells (B, C).

*L. casei *surface-displayed expression of VP4 and VP4-LTB, respectively, was confirmed by immunofluorescence. Overnight cultures of pPG612.1-VP4 and pPG612.1-VP4-LTB were grown in basal MRS medium supplemented with either xylose or glucose. The cells were washed, incubated with mouse anti-VP4 anti serum followed by a FITC-conjugated goat anti-mouse IgG. VP4 was detected on the surface of pPG612.1-VP4 and pPG612.1-VP4-LTB cells grown in the presence of xylose (Figure 3B and 3C). No immunofluorescence was observed when wild-type *L. casei *393 was incubated in a similar fashion (cells were stained red by Evans blue dye, Figure 3A).

### Antibody responses following oral immunizations

**Figure 4:**
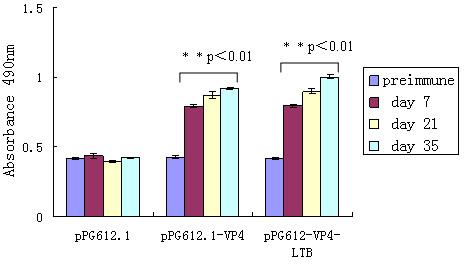
**Specifis IgG antibodies in serum**. Serum from groups of mice (10 mice every group) immunized orally with pPG612.1-VP4, pPG612.1-VP4-LTB and equivalent dose of pPG612.1 were analyzed for the presence of anti-VP4 specific IgG by ELISA. IgG titers of serum in mice given pPG612.1-VP4 or pPG612.1-VP4-LTB were similar but higher than that of mice given pPG612.1. ** P < 0.01 significant difference between IgG titers of serum in mice given pPG612.1-VP4 and pPG612.1 on day 7, 21 and 35. Results are the IgG titers ± standard errors of the means in each group.

The ability of the respective VP4-expressing *L. casei *vectors to elicit systemic and/or mucosal immunity was assessed by determining the presence of anti-VP4 IgG and IgA antibodies, respectively. Anti-VP4 IgG antibody levels in serum of mice treated with either pPG612.1-VP4 or pPG612.1-VP4-LTB were similar to each other but higher than only with pPG612.1 (Figure 4). After the first booster, a prompter and stronger level of anti-VP4-specific serum IgG was elicited in mice that were administered with recombinant strains. A statistically significant difference was observed on day 7, 21 and 35 (** P < 0.01, Figure 4). No significant elicitation of anti-VP4 antibodies was observed in the control groups that received pPG612.1.

**Figure 5:**
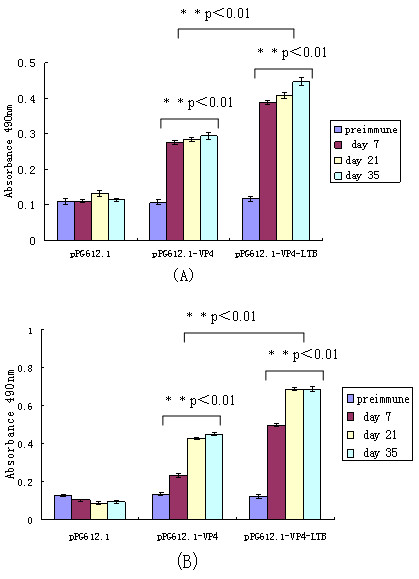
**Specific IgA levels in ophthalmic and vaginal wash were detected after oral immunization (10 mice every group administered with different recombinant strains)**. Specific IgA antibody titers were detectable in the mice immuned with pPG612.1-VP4 and pPG612.1-VP4-LTB after the first administration (Fig. 5A, B). Statistically significant difference (** P < 0.01) was observed in ophthalmic and vaginal wash of mice administered with recombinant strains after seven days. IgA levels elicited by pPG612.1-VP4-LTB were higher than those elicited following pPG612.1-VP4 immunization and the difference is significant statistically (** P < 0.01). Bars represent the IgA titers ± standard errors of the means in each group.

**Figure 6:**
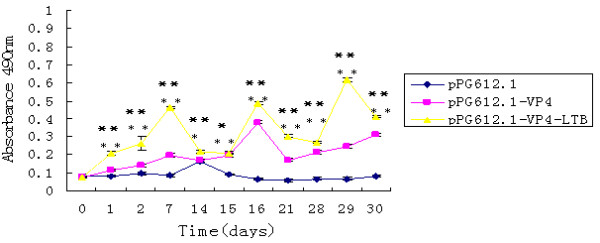
**Specific IgA levels in fecal pellets after oral immunization**. The mice (10 every group) received three consecutive immunization, three times at 2-week intervals. The control group of mice received the same dose of pPG612.1. Fecal pellets were collected 1, 2, and 7 days after every immunization. Both of the groups immuned with pPG612.1-VP4 or pPG612.1-VP4-LTB produced specific IgA. Statistically significant difference (** P < 0.01) was observed in fecal pellets of mice administered with recombinant strains after one day. The levels of IgA in fecal pellets induced by pPG612.1-VP4 appeared lower than those induced by pPG612.1-VP4-LTB (*P < 0.05,**P < 0.01). Results are the IgA titers ± standard errors of the means in each group.

As the results showed, there were no substantial differences in mucosal IgA levels between experimental and control groups prior to oral immunization. Following administration with the *L. casei *recombinants, specific anti-VP4 mucosal IgA responses were observed. After the second boost, significant levels of anti-VP4 IgA were observed from mucosal secretions following administration of either pPG612.1-VP4 or pPG612.1-VP4-LTB compared to responses observed in control mice. Statistically significant difference (** P < 0.01, Figure 5 and 6) was observed in ophthalmic and vaginal wash of mice administered with recombinant strains after seven days and fecal pellets after one day. The mucosal IgA levels elicited by pPG612.1-VP4-LTB were higher than pPG612.1-VP4 immunization and the difference is significant statistically (* P < 0.05,* *P < 0.01, Figure 5 and 6). This indicated that LTB enhanced the mucosal immune system response.

### Neutralization ability of the induced antibodies analysis

**Figure 7:**
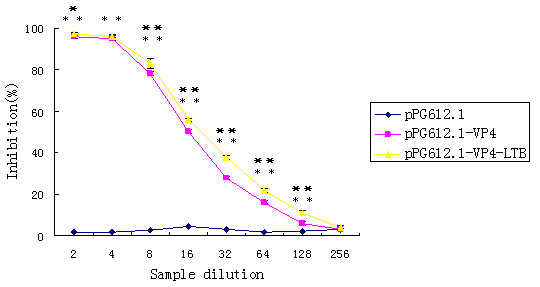
**Neutralization ability of the sera prepared from mice immunized with pPG612.1-VP4 and pPG612.1-VP4-LTB**. The maximum reduction of CPE, expressed as a percentage of CPE obtained for the negative control samples, by using sera collected from mice fed with pPG612.1-VP4 or pPG612.1-VP4-LTB, was 50.28% ± 0.83% or 56.06% ± 0.77%, respectively. Statistically significant difference (** P < 0.01) was observed in sera of mice administered with recombinant strains. The neutralizing efficacy of anti-VP4 IgG from mice immunized with pPG612.1-VP4 was lower than antibodies obtained from mice immunized with pPG612.1-VP4-LTB and the difference was significant statistically (* P < 0.05,**P < 0.01). Results are mean values and standard errors (error bars) of triplicates.

The Neutralization ability of the induced antibodies was investigated to further detect whether the antibody responses were against RV. Results demonstrated that the presence of anti-rPRV-VP4 IgG in the culture medium conferred statistically significant neutralizing effects (** P < 0.01, Figure. 7) on RV infection. A near 50.28% ± 0.83% reduction of CPE was consistently observed when the assays were carried out using 2-to 16-fold diluted sera from mice immunized with pPG612.1-VP4, and a 56.06% ± 0.77% reduction of CPE was observed by using 2-to 16-fold diluted sera from mice immunized with pPG612.1-VP4-LTB. The inhibitory effect decreased gradually on further dilutions of sera and reached to the level similar to that of the control, which of sera administered with pPG612.1-VP4 is 1:128 and pPG612.1-VP4-LTB is 1:256 in Figure. 7. The neutralizing efficacy of anti-VP4 IgG from mice immunized with pPG612.1-VP4 was lower than pPG612.1-VP4-LTB and the difference was significant statistically (*P < 0.05,* *P < 0.01, Figure. 7).

## Discussion

Porcine rotaviruses are the major cause of acute diarrhea in the piglets and can cause mild to severe diarrhea with potentially high morbidity and mortality rates. Infection with porcine rotavirus has been an economic concern to worldwide pig breeders. Vaccination is the main prophylatic method for the prevention of porcine rotavirus infections. Mucosal immunization offer a number of advantages over other routes of antigen delivery, including ease of administration, cost effectiveness and the capacity of inducing both local and systemic immune responses [36-41].

To assess mucosal immune responses, specific IgA anti-VP4 protein levels were examined from various mucosal surfaces. Oral administration of recombinant VP4 or VP4-LTB-expressing *L. casei *induced both systemic (IgG) and mucosal (IgA) immune responses. Specifically, IgA specific for VP4 could be isolated from the gastrointestinal tract, vagina and eye secretions compared to no detectable IgA anti-VP4 responses in control animals. These experiments suggested that *L. casei *expressing recombinant VP4 could be used in the vaccination of pigs, potentially protecting them from porcine rotavirus infections since this vector successfully elicited a significant and specific anti-VP4 IgA response.

The titers of anti-VP4 IgG in the serum from mice immunized with the *L. casei *pPG612.1-VP4 or pPG612.1-VP4-LTB were similar but higher than the control group. rLc393:pPG612.1-VP4-LTB induced even higher IgA specific for VP4 compared to mice immunized with the pPG612.1-VP4 as a result of the LTB mucosal adjuvant. It demonstrated the specific mucosal adjuvanticity of LTB, highlighting its potential use as a safe and effective mucosal adjuvant that can be used in conjunction with VP4 for the elicitation of specific anti-porcine rotavirus immunity.

Furthermore, in order to confirm the efficacy of the induced antibodies in inhibiting the virus, we tested whether sera collected from immunized mice could inhibit the infection of RV in MA104 cells by neutralization ability assay. The results showed that serum collected from mice immunized with recombinant strains demonstrated statistically significant inhibition. The neutralization by sera antibodies obtained from mice immunized with pPG612.1-VP4-LTB was more effective than that of mice immuned with the pPG612.1-VP4.

## Conclusion

In this report, we described the methods for constructing two *L. casei *recombinant expression vectors expressing the porcine rotavirus VP4 antigen or VP4-LTB fusion protein. *L. casei *is an excellent delivery vector since it can withstand the rigors of the intestinal environment in addition to being able to colonize different mucosal sites (gastrointestinal and genital tracts) and guaranteeing proper (intact) presentation of the respective antigens to the immune system. In addition to the versatility of *L. casei*, it possesses probiotic properties making it an even more attractive vaccine delivery system *i.e*., immunization with *L. casei *expressing VP4-LTB elicited potent anti-VP4 IgA responses. Testing the efficacy in a porcine vaccination and infection model is a next step in testing the efficacy of this vaccine formulation.

## Methods

### Strains and culture conditions

*L. casei *ATCC 393 (a kind gift of Jos Seegers, NIZO, The Netherlands) was grown anaerobically in MRS broth (Sigma, St, Louis, MO) at 37°C without shaking. To analyze protein expression, transformed *L. casei *were grown in basal MRS medium (10 g peptone, 8 g beef extract, 4 g yeast extract, 2 g potassium phosphate, 5 g sodium acetate, 1 ml Tween 80, 2 g diammonium citrate, 0.2 g magnesium sulfate, and 0.05 g manganese sulfate per liter) supplemented with 2% xylose. *L. casei *was plated on MRS medium with 1.5% agar. The antibiotic concentration used for the selection of lactobacilli transformants was 10 μg/ml of chloromycetin (Cm; Sigma). Porcine rotavirus JL94 (belonging to P[7]) was conserved in the laboratory.

### Mice

Balb/c mice (female) weighing 25-30 g (7 weeks of age) were obtained from the inbred colony maintained at the Harbin Veterinary Research Institute. Each experimental and control group consisted of 10 mice. The animals were fed balanced rodent food and water *ad libitum*. The mice were handled and maintained under strict ethical conditions according to the international recommendations for animal welfare and the Ethical Committee for animals sciences of HeiLongJiang province (032/2006).

### Mouse anti-VP4 antibodies

The mouse anti-VP4 antibodies used in Western-blot and immunofluorescence analysis had been prepared and stored in our laboratory. The recombinant plasmid VP4-pGEX-6P-1 was constructed and transformed into E. coli BL21(Yan Song). The recombinant strain was induced with IPTG. The serum was obtained from the Balb/c mice immunized with the purified VP4 protein. Western-blot test and neutralization test circumstantiate the expressed protein has biological activity(data not shown).

### Expression plasmid construction

**Figure 8:**
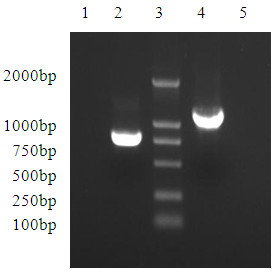
**Target amplification fragments of VP4 and VP4-LTB fusion gene**. Lane 1,5: Blank controls; Lanes 2: Target amplification fragment of VP4 gene; Lanes 3: 2000 bp DNA marker; Lane 4:Target amplification fragment of VP4-LTB fusion gene.

The pPG612.1 plasmid is an expression vector containing an ssUsp signal peptide secretion sequence (kindly supplied by Jos Seegers, NIZO, The Netherlands). Nucleic acid manipulation and cloning procedures were performed according to standard procedures [42]. All DNA manipulations were performed according to standard procedures [43]. A gene fragment about 756 bp (VP8) encoding the main structural polypeptide of VP4 (obtained from the genome of PRV strain JL94) was amplified by polymerase chain reaction (PCR) using forward primer 5'-CAGGGATCCAATGGCTTCGCTCA-3'(*BamH*I site underlined) and the reverse primer 5'-GGCCTCGAGAGCTCTTGTGTGCA-3'(*Xho*I site underlined) (Figure 8). PCR amplification conditions were as follows: 95°C, 5 min followed by 30 cycles at 94°C, 1 min; 56.5°C, 1 min; 72°C, 1 min and a 72°C 10 min final extension. The VP4 gene PCR product was cleaved with *BamH*I and *Xho*I and ligated into the corresponding sites of pPG612.1 digested with *BamH*I and *Xho*I, respectively, giving rise to pPG612.1-VP4. A gene fragment of about 375 bp encoding the *E. coli *LTB structural polypeptide was amplified by PCR using the forward primer 5'-AAGGTCGACTGCTGTVVGATGAATAAAGTAAAATGTTAT-3' (*Sal*I site underlined) and the reverse primer 5'-AAGCTCGAGCTAGTTTTCCATACTGATTGCCG-3'(*Xho*I site underlined). PCR amplification conditions were as follows: 95°C, 5 min followed by 30 cycles of 1 min at 94°C; 1 min, 56°C; 1 min, 72°C and a final extension at 72°C for 10 min. The LTB PCR product was cleaved with *Sal*I and *Xho*I and inserted into the corresponding sites in pPG612.1-VP4 digested with *Sal*I and *Xho*I, giving rise to pPG612.1-VP4-LTB (Figure 8).

Electroporation of *L. casei *was carried out as previously described [44]. Briefly, plasmid DNA (10 μl) was added to 150 μl of *L. casei *393, gently mixed at 4°C for 5 min and subjected to a single electric pulse (25 μF of 2.5 kV/cm). The mix was then incubated in MRS medium without Cm at 37°C anaerobically for 2 h. Recombinant strains were selected on MRS-agar medium containing 10 μg/ml of Cm. The sequences of the respective *L. casei *393 transformants were confirmed by plasmid DNA sequencing.

### Protein expression and Western-blot analysis

To analyze the expression of the VP4 and VP4-LTB fusion protein following xylose induction of rLc393:pPG612.1-VP4 and pPG612.1-VP4-LTB, respectively, overnight cultures grown in basal MRS broth supplemented with xylose (or glucose as a negative induction control) and pellets collected by centrifugation at 12,000 × g for 10 min. The pellets were washed twice with sterile 50 mM Tris-Cl, pH 8.0 and treated with 10 mg/ml lysozyme at 37°C for 60 min. The lysates were centrifuged at 12000 × g for 10 min and subjected to 10% sodium dodecyl sulphate polyacrylamide gel electrophoresis (SDS-PAGE) and either stained with Coomassie blue or electrotransferred onto nitrocellulose membranes. The immunoblots were blocked with PBS containing 5% skimmed milk for 2 hr at 37°C. Blots were washed three times between all steps for ten minutes. Blots were incubated with 1:800 dilution(100 μL) of mouse anti-VP4 antibodies in phosphate-buffered saline (PBS), washed and then probed with a horseradish peroxidase (HRP)-conjugated goat anti-mouse IgG (Sigma) diluted at 1:2500(100 μL) in PBS. The blots were washed and incubated with the Chemiluminescent Substrate reagent (Pierce, Rockford, IL) according to the manufacturer's instruction. Control blots incubated with secondary antibody only did not result in visible protein band reactivity.

### Immunofluorescence analysis

Immunofluorescence was used to analyze VP4 and VP4-LTB protein surface expression by either rLc393:pPG612.1-VP4 or pPG612.1-VP4-LTB as described previously [45]. Briefly, 2 ml induced cultures were harvested to an OD600 = 0.5-0.6 and then resuspended in 1 ml sterile PBS 3% bovine serum albumin (BSA) containing anti-VP4 antibodies and then incubated overnight at 37°C. The cells were then pelleted, washed 3 times with sterile PBS 0.05% Tween 20. The cell-antibody complexes were then incubated for 6 h at 37°C in the dark with fluoreoscein isothiocyanate (FITC)-conjugated goat anti-mouse IgG (Sigma) containing 1% Evans blue. Cells were washed 3 times with PBS 0.05%, Tween 20 and then air-dried on a glass slide. Analysis was performed using a confocal microscope. Non-induced or glucose-induced recombinant strains were used as negative controls.

### Immunizations

rLc393:pPG612.1-VP4 and rLc393:pPG612.1-VP4-LTB were cultured and centrifuged as described above. Cell pellets were washed once with sterile PBS and resuspended in PBS (pH 7.4). Mice were orally vaccinated with 0.2 ml 10^9 ^colony-forming units (c.f.u.)/ml of the recombinant strains, respectively. A control group of 10 mice received *L. casei *ATCC 393 containing the empty plasmid was also included. Mice in all groups were immunized on days 0, 1 and 2 and boosted on days 14, 15 and 16 and again on days 28, 29 and 30.

### Enzyme-linked immunosorbent assay (ELISA)

Mouse serum was collected on days 7,14,21 and examined for specific anti-VP4 antibodies by ELISA. Feces was collected at 1, 2 and 7 days after every immunization as described previously [46]. Ophthalmic washes were obtained by washing the eyes with 50 μl PBS 7 days after every immunization. Vaginal washes were collected by washing the vagina with 200 μl PBS 7 days after every immunization. All samples were stored at -20°C until assayed by ELISA.

Polystyrene microtitre plates were coated overnight at 4°C with either porcine rotavirus propagated on MA104 cells or with supernatants harvested from MA104 cells cultured without rotavirus as negative control. ELISA plates were washed 3 times with PBS 1%Tween 20 and then blocked with PBS 5% skim milk at 37°C for 2 h. Serum or mucosal wash samples were serially diluted in PBS 1% BSA and incubated at 37°C for 1 h, washed 3 times and then incubated with a 1:2000 dilution(100 μL) of an HRP-conjugated goat anti-mouse IgA (Sigma) or IgG (Sigma), washed and visualized following the addition of 100 μl of o-phenylene diamine dihydrochloride substrate(Sigma). The absorbance was measured at 490 nm. Differences in the samples between treatments were examined for the level of significance by ANOVA.

### Neutralization ability of the induced antibodies

Serum samples from mice immunized with recombinant strains expressing VP4 or VP4-LTB were evaluated [47] to determine the neutralization ability of the induced antibodies. In brief, sera from mice fed with non-expressor strains was used as negative control. Fifty microliters of samples in serial dilutions (from 1:2 to 1:512) was prepared in a 96-cell plate. RV adjusted to 200 TCID50 in 50 μL of virus diluent (10% concentrated Hanks balanced salt solution, pH 7.4) was added to the cell plate containing serially diluted serum. The mixture of antibody and virus was mixed and incubated at 37°C for 1 h. Then 100 μL of MA104 cells (used for virus infection) was added to the antibody-virus mixture and incubated in a 5% CO2 incubator at 37°C for 5 days. The overlay medium was then discarded, after which the wells were washed three times with sterile PBS, pH 7.4, and stained with 1% crystal violet solution. Differences in the number of plaques formed between treatments were examined for the level of significance by ANOVA.

### Statistical analysis

Statistical significance was determined using ANOVA, with a *P *value < 0.05 considered as significant.

## Authors' contributions

XQ carried out construction of expression plasmid, participated in the sequence alignment and drafted the manuscript. GL carried out the protein expression and immunoassays. XW and XL carried out the Immunizations. ML performed the statistical analysis. YL conceived of the study, and participated in its design and coordination. All authors read and approved the final manuscript.
